# Epigenetic Regulation of Epithelial to Mesenchymal Transition in the Cancer Metastatic Cascade: Implications for Cancer Therapy

**DOI:** 10.3389/fonc.2021.657546

**Published:** 2021-04-29

**Authors:** Qiu-Luo Liu, Maochao Luo, Canhua Huang, Hai-Ning Chen, Zong-Guang Zhou

**Affiliations:** ^1^ Department of Gastrointestinal Surgery, State Key Laboratory of Biotherapy and Cancer Center, West China Hospital, Sichuan University, Collaborative Innovation Center for Biotherapy, Chengdu, China; ^2^ Department of Biotherapy, State Key Laboratory of Biotherapy and Cancer Center, West China Hospital, and West China School of Basic Medical Sciences & Forensic Medicine, Sichuan University, and Collaborative Innovation Center for Biotherapy, Chengdu, China

**Keywords:** epithelial to mesenchymal transition, epithelial–mesenchymal plasticity, epigenetics, metastatic cascade, cancer therapy

## Abstract

Metastasis is the end stage of cancer progression and the direct cause of most cancer-related deaths. The spreading of cancer cells from the primary site to distant organs is a multistep process known as the metastatic cascade, including local invasion, intravasation, survival in the circulation, extravasation, and colonization. Each of these steps is driven by the acquisition of genetic and/or epigenetic alterations within cancer cells, leading to subsequent transformation of metastatic cells. Epithelial–mesenchymal transition (EMT), a cellular process mediating the conversion of cell from epithelial to mesenchymal phenotype, and its reverse transformation, termed mesenchymal–epithelial transition (MET), together endow metastatic cells with traits needed to generate overt metastases in different scenarios. The dynamic shift between these two phenotypes and their transitional state, termed partial EMT, emphasizes the plasticity of EMT. Recent advances attributed this plasticity to epigenetic regulation, which has implications for the therapeutic targeting of cancer metastasis. In this review, we will discuss the association between epigenetic events and the multifaceted nature of EMT, which may provide insights into the steps of the cancer metastatic cascade.

## Introduction

Metastasis is a complex process by which tumor cells spread to distant sites and is the leading cause of mortality among patients with cancer (1). To successfully spawn a metastasis, cancer cells need to adopt invasive properties to migrate locally, disseminate and survive in the circulation, and acquire initiating capabilities to form colonization at distant organs, which is termed as the metastatic cascade (2). During metastasis, cancer cells have been proposed to hijack the epithelial–mesenchymal transition (EMT), a transient change of cell phenotype from the epithelial to mesenchymal state initially characterized by embryonic development (3).

Cancer cells with the epithelial phenotype display apical–basal polarity, specialized intercellular junctions, and contact with the basement membrane (3). These differentiated cells are shown to be in the state of proliferation and drug sensitivity (4). After EMT initiation, cancer cells may dedifferentiate into the mesenchymal state, leading to increased motility and development of an invasive phenotype (5). Cancer cells with the mesenchymal state have stem cell-like characteristics and are more resistant to treatment (4). However, this subtype of cells exhibits low levels of proliferation (6). In other words, the mesenchymal cells are predominant in the context of dissociation from the primary tumor, whereas cells in the epithelial state are the superior subpopulation during the outgrowth of both primary and secondary tumors.

The involvement of EMT in metastasis and even the very existence of this process has been a subject of debate for a long time. This is in large part due to the technical challenge and poor understanding of EMT plasticity. The detection of EMT *in vivo* is difficult owing to the transient and reversible nature of this process and tumor cells that have undergone EMT are indiscernible from stromal cells in the tumor microenvironment (7). Despite these challenges, recent advances in lineage marking and intravital real-time visualization techniques have enabled direct characterization of EMT during cancer progression (8–10).

Nevertheless, a large number of studies forcing downregulation or overexpression of key EMT-TFs have reported contradicting roles of EMT in forming metastasis (4, 6). Of note, there were two studies employing the same pancreatic carcinoma model. While one study indicated that EMT program suppression by genetically knock out of *SNAI1* or *TWIST* failed to alter the emergence of metastasis (11), the other one showed that EMT suppression by *ZEB1* depletion significantly repressed the spread to distant organs (12). An extrapolation can be made that forcibly fixing cells in either a mesenchymal or epithelial state may lead to the loss of phenotypic plasticity, which in turn results in the failure of metastatic outgrowth. Besides, evidence from the past few years has demonstrated that tumor cells rarely undergo a full EMT, instead, most of them proceed to a state with certain mesenchymal characteristics as well as some epithelial qualities, namely partial EMT (13, 14). This adoption of hybrid epithelial/mesenchymal features, together with the ability to move along the epithelial/mesenchymal spectrum has now been termed as epithelial–mesenchymal plasticity (EMP) (15).

According to the previous understanding, cancer cells that have undergone EMT were fixed in a specific state. This notion is contrasted by recent evidences on the phenotypic reversal of EMT after arriving at distant sites, where contextual signals originating from the microenvironment induce a mesenchymal–epithelial transition (MET) program to drive disseminated cells to revert to the epithelial state (10, 16, 17). The dynamic process of EMT and MET endows tumor cells with multiple qualities that are required to complete all the steps of metastatic cascade (18). The global reprogramming of gene expression involved in EMP, combined with non-genetic heterogeneity displayed by clonal populations, implying that reversible epigenetic regulation rather than permanent genetic mutations as potential molecular mechanisms underlying this process (4, 19). Emerging evidence has revealed the functional roles of epigenetic mechanisms including histone modification, DNA methylation, and non-coding RNA in EMT/EMP that facilitate cancer metastasis (20).

In this review, we will discuss the epigenetic changes involved in the regulation of EMT in metastatic cascade and emphasize herein the difficulties and controversies of epigenetic therapies that target EMT for cancer treatment, with the aim to provide a perspective for future research.

## Epigenetic Regulation of EMT in the Metastatic Cascade

### EMT Initiation During Local Invasion

**Figure 1:**
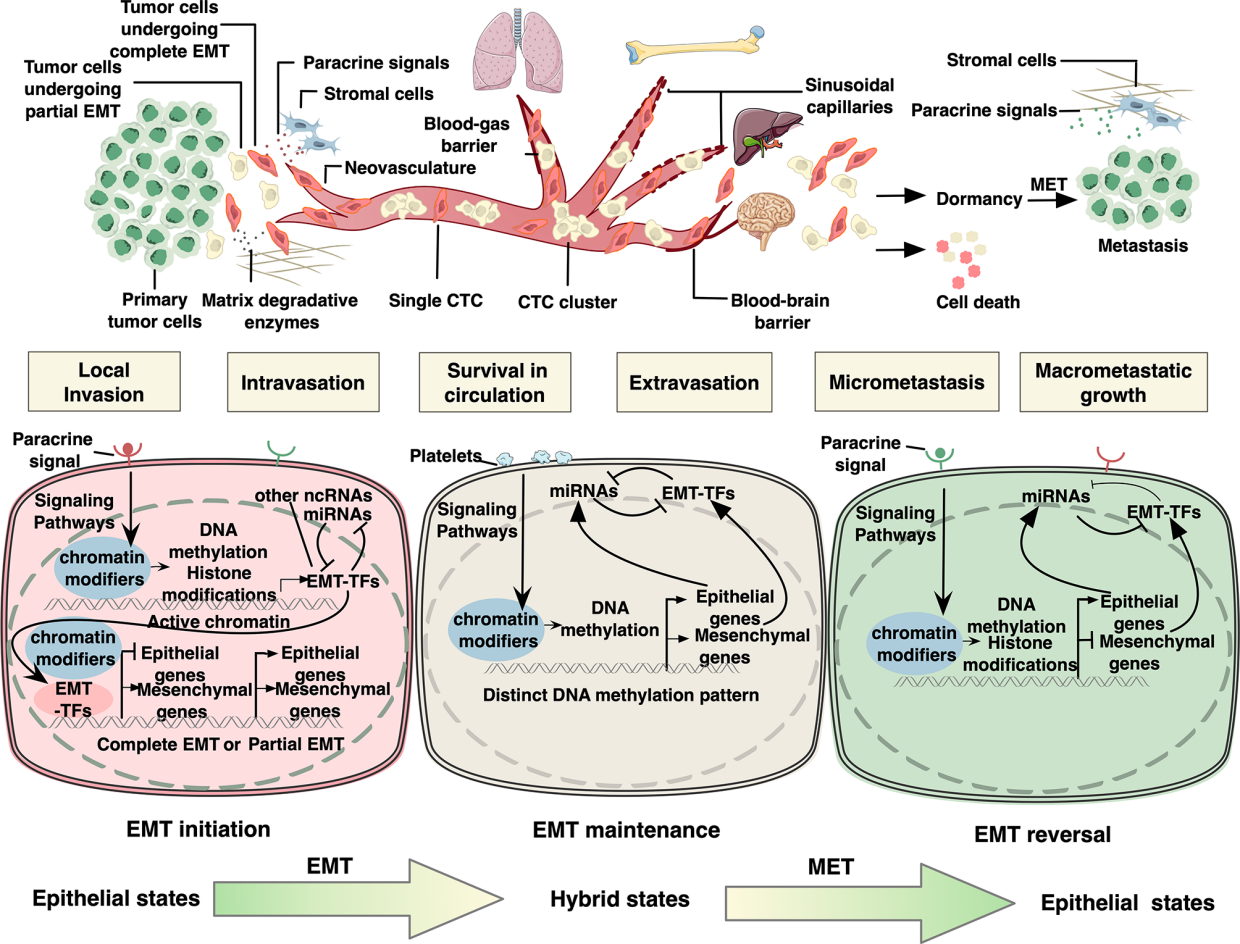
Epigenetic regulation of epithelial to mesenchymal transition in cancer metastatic cascade. In the primary tumor, for local migration, epithelial tumor cells initiate EMT program by activating signaling pathways responsive to specific environmental stimuli. As a result, EMT-related transcription factors (EMT-TFs) are induced, whose expression can be regulated by DNA methylation, histone modifications, and RNA-mediated epigenetic regulation. Next, EMT-TFs collaborate with various epigenetic proteins to regulate the expression of downstream EMT effectors. It is assumed that tumor cells undergo partial EMT instead of complete EMT to improve their metastatic ability. After local migration, tumor cells disseminate into the circulation, known as circulating tumor cells (CTCs). CTCs interact with platelets through different cell surface receptors and ligands, by which they sustain EMT *via* platelet-derived TGFβ signals in the absence of stroma-derived signaling cues that initiated EMT in the microenvironment of the primary lesion. CTC clusters and single CTCs have been demonstrated to exhibit distinct DNA methylation patterns of EMT-associated genes. In addition, members of miR-200 family have been acknowledged as key regulators of EMT maintenance in CTCs by forming double-negative feedback loops with EMT TFs, in which they reciprocally repress each other’s translation or transcription. The extravasation mechanisms of CTCs vary widely depending on the flow direction and organ-specific vascular structure. Upon reaching the distant sites, the vast majority of carcinoma cells will not survive, while a small minority persist in a quiescence state as dormant cancer cells until reinitiate growth and form colonization. It is proposed that invasive cells need to undergo MET to acquire the capability of macrometastatic growth. MET is mainly triggered and regulated by contextual signals from microenvironment or due to the absence of EMT-inducing signals from the primary tumor microenvironment, resulting in downregulation of several EMT-TFs. The negative feedback loops consisting of pairs of miRNAs and EMT-TFs are also considered the driving force underpinning this state reversibility. Both EMT-TFs and micro RNAs in these loops have also been shown to be elaborately regulated by epigenetic mechanisms including DNA methylation and histone modifications.

For local migration, epithelial cells must acquire traits that favor detachment from the primary sites and penetration into the adjacent parenchyma (**Figure 1**). In the primary tumor, epithelial cell junctions including tight junctions, adherens junctions, desmosomes, gap junctions, and hemidesmosomes are formed to maintain cell–cell and cell–matrix contacts. Upon EMT, cell–cell junctions (mainly E-cadherin) are destabilized to allow cell detachment, while cell–matrix contacts mediated by integrins are also destructed and switched to specific patterns that promote cell migration (21). EMT activation also leads to upregulation of extracellular proteases such as matrix metalloproteinases (MMPs) and serine proteinases, which not only degrades extracellular matrix (ECM) but also cleaves E-cadherin and releases bioactive fragments that promote cell motility (2). Furthermore, EMT program results in actin cytoskeleton remodeling, forming actin-rich protrusions on the plasma membrane called lamellipodia, filopodia, and invadopodia, in which invadopodia plays a key role in cell invasion as it may also degrade ECM (3).

**Figure 2:**
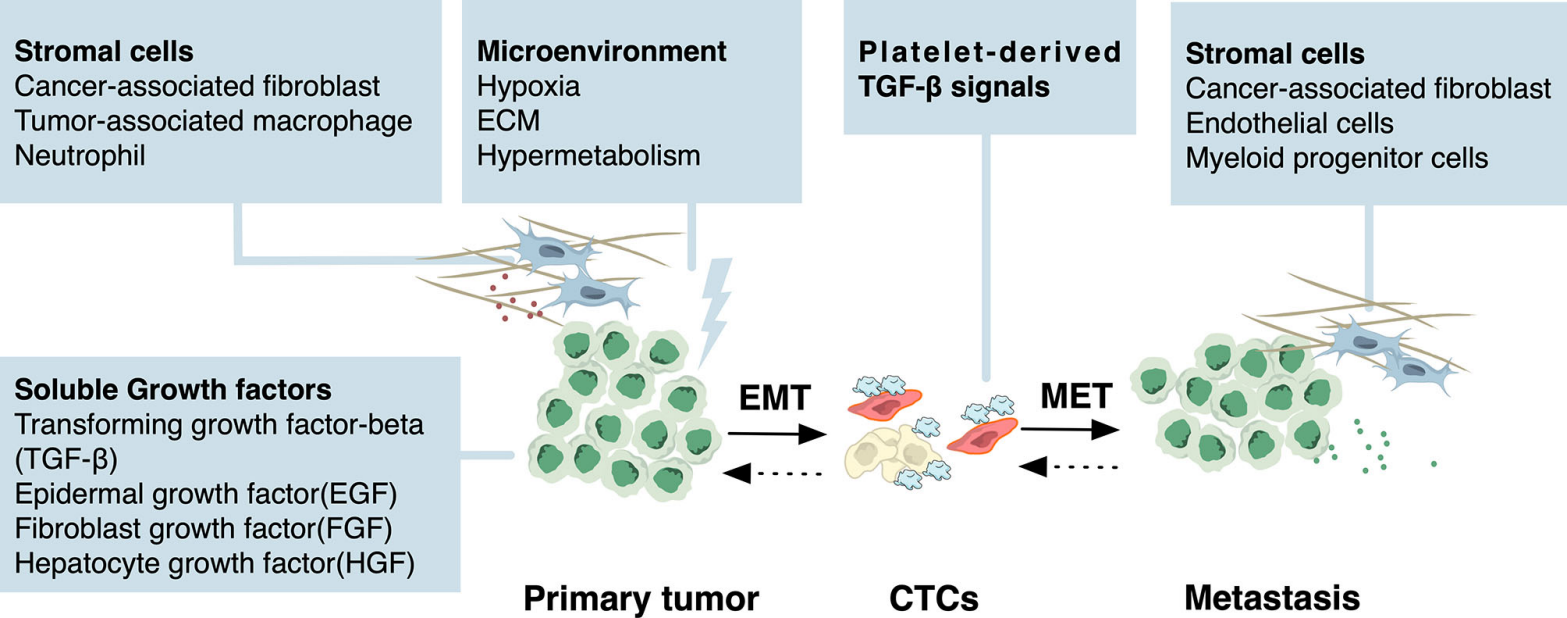
Environmental stimuli of EMT and MET. Specific environmental stimuli lead to the initiation of EMT program during cancer development and progression. In the microenvironment of the primary lesion, soluble growth factors including transforming growth factor beta (TGF-β), epidermal growth factor (EGF), fibroblast growth factor (FGF), hepatocyte growth factor (HGF) secreted by stromal cells such as cancer-associated fibroblast, tumor-associated macrophage, and neutrophil activate several signaling pathways in cancer cells. These signaling pathways then regulate the EMT-related transcription factors (EMT-TFs) through epigenetic mechanisms. Hypoxic stress and altered metabolism from the microenvironment are also stimuli of EMT. For CTCs, in the absence of stroma-derived signaling cues that initiated EMT, platelet-derived TGF-β signals serve as the stimuli to sustain EMT. Although the external stimuli of MET are currently largely unknown, it has been proposed that MET can be triggered by stromal cells from the microenvironment including cancer-associated fibroblast, endothelial cells, and myeloid progenitor cells, or passively result from the absence of EMT-inducing signals from the primary tumor microenvironment.

**Figure 3:**
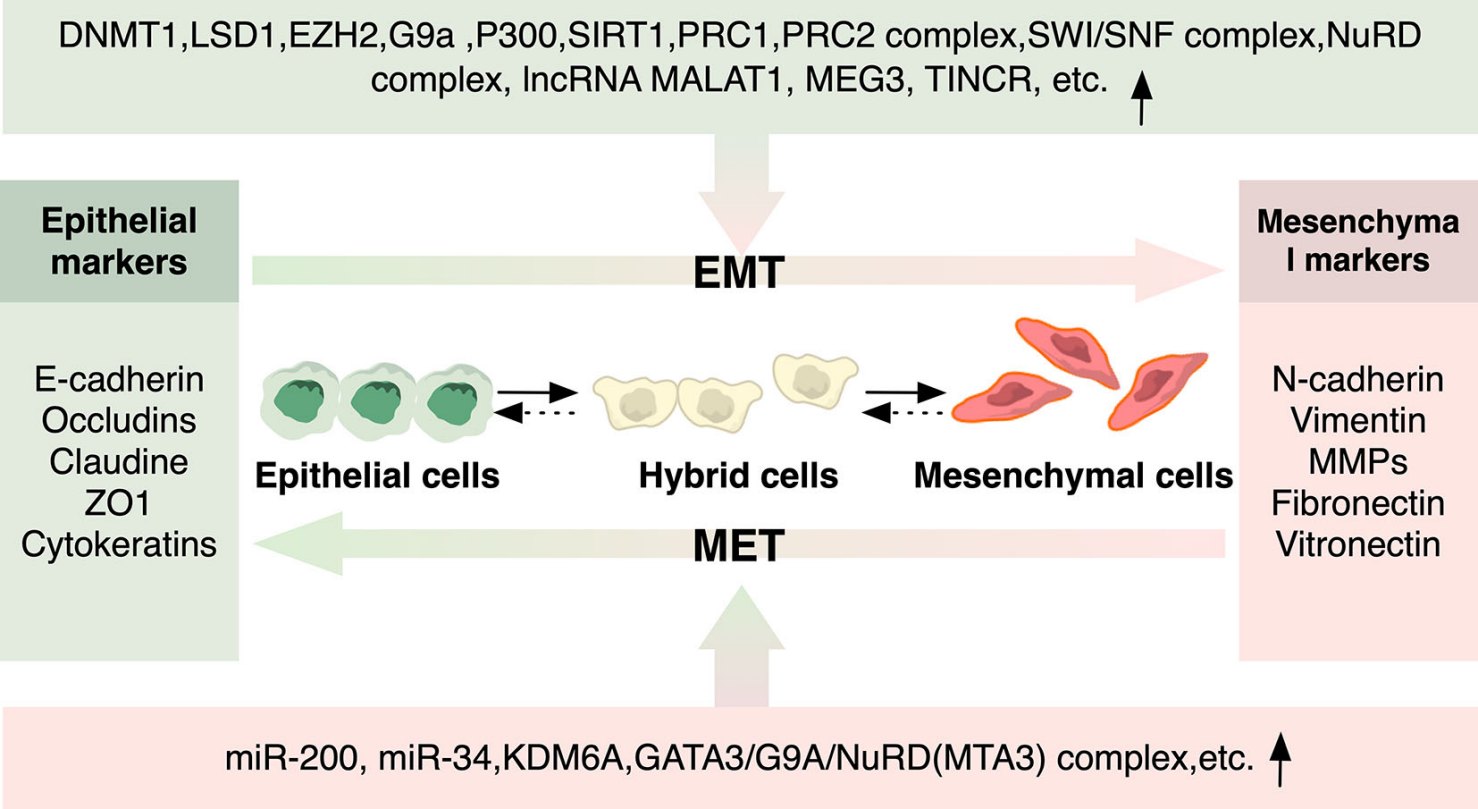
Epigenetic Regulators of EMT and MET. Epigenetic modulators involved in histone modification, DNA methylation, and non-coding RNA in EMT/MET that regulate cancer metastasis are depicted. Epigenetic regulators can either be recruited by EMT-TFs to regulate the expression of downstream EMT-associated genes, or they can directly function as either transcriptional suppressor or activators of EMT-TFs as well as downstream EMT-associated genes.

The initiation of EMT program is considered responsive to specific environmental stimuli during cancer development and progression (7) (**Figure 2**). Soluble growth factors including transforming growth factor-beta (TGF-β), epidermal growth factor (EGF), fibroblast growth factor (FGF), hepatocyte growth factor (HGF) secreted by stromal cells such as cancer-associated fibroblast, tumor-associated macrophage and neutrophil play a critical role in activating EMT in cancer cells (18). Subsequently, activated signaling such as TGFβ-SMAD3, WNT-β catenin, and Notch induces the expression of EMT-related transcription factors (EMT-TFs) such as Zeb1, Zeb2, Snail, Slug, and Twist. These EMT-TFs act in concert to downregulate the expression of epithelial markers such as E-cadherin, claudins, and occludin, and concurrently upregulate the expression of mesenchymal markers such as N-cadherin, vimentin, and fibronectin (3). Mechanistically, these EMT-TFs are regulated at a transcriptional level by DNA methylation, histone modifications, and RNA-mediated epigenetic regulation (6, 22) (**Figure 3**). For example, in breast cancer, the GATA3/G9A/NuRD(MTA3) complex inhibited the expression of Zeb2 by catalyzing H3K9 methylation of *ZEB2* promoter (23), while Snail suppressed the expression of E-cadherin by recruiting histone lysine-specific demethylase 1 (LSD1) to *CDH1* promoter (24). In addition, other chromatin modifiers, including DNA methyltransferases (DNMTs) (25), histone methyltransferases (HTMs) (26), histone acetyltransferases (HATs) and histone deacetylases (HDACs) (27, 28), chromatin-remodeling complexes such as PRC1 (29) and SWI/SNF (30), have been implicated in the interplay with EMT-TFs, functioning as either transcriptional suppressor or activators of EMT-TFs as well as downstream EMT-associated genes in multiple cancer types. Furthermore, epigenetic profiling has revealed genome-scale reprogramming during EMT, including alterations in DNA methylation in ovarian cancer cells (31) and an increase in H3K4Me3 and H3K36Me3 while a reduction in H3K9Me2 in mouse hepatocytes (32). Recent research discovered a more unifying mechanism underlying EMT in pancreatic cancer cells, in which a genome-wide increase in H3K36me2 modulated the expression of nearly all master EMT-TFs (33).

Notably, epigenetic mechanism was also shown to be involved in partial EMT, where inactive EZH2 in FAT1-mutated skin squamous cell carcinoma and lung cancer cells reduced the inhibitory action of H3K27me3 on the promoter of *SOX2*, contributing to the epithelial phenotype of cancer cells in hybrid EMT state (34). Additionally, RNA-mediated epigenetic regulation also has an impact on EMT program. For instance, members of miR-200 and miR-34 family were acknowledged as key regulators of EMT in a variety of cancers by forming double-negative feedback loops with Zeb and Snail family, in which they reciprocally repressed each other’s translation or transcription (35, 36). However, although frequently regarded as EMT inhibitors, the expression level of miR-200 family can be elevated in several types of cancer, in which its overexpression is correlated with poor clinical outcome (37, 38).

### EMT Maintenance During Intravasation, Survival in Circulation and Extravasation

#### Intravasation and Survival in Circulation

After dissociating from the primary tumor, tumor cells must acquire the ability of transendothelial migration to get access to the systemic circulation. EMT promotes intravasation *via* orchestrating several biological processes including angiogenesis and disruption of the endothelial integrity (**Figure 1**). During metastasis, abnormal neovasculature with high density and hyperpermeability lead to efficient access to the bloodstream. In breast cancer, EMT contributes to the form of a conduit for metastatic dissemination as evidenced by upregulated angiogenesis genes including *VEGFA* and higher microvascular density displayed by both primary and metastatic tumors upon EMT (39). In addition to angiogenesis, EMT is also required during intravasation. For instance, in breast cancer, Snail1 induced MT1-MMP and MT2-MMP and enhanced disruption of the basement membrane (40). Although the specific mechanism by which EMT-mediated MMPs regulate intravasation has not been fully elucidated, MMPs-dependent intravasation has already been experimentally addressed in other studies (40–42), suggesting the potential role for EMT in facilitating transendothelial migration.

Tumor cells disseminating into the circulation, which are termed as circulating tumor cells (CTCs), may form cell clusters by associating with each other or other cells such as platelets to survive under the immune system and shear stress (**Figure 1**). Results from previous studies demonstrated that CTCs exhibit various states, including mesenchymal, epithelial, and hybrid states. Along this line, it has been proposed that a complete transition to mesenchymal states is associated with single cell migration, while the hybrid states with transient cell-cell junctions resulting from partial EMT facilitate collective migration (43). Of note, CTC clusters in hybrid state are shown to have up to 50-fold enhanced dissemination potential than single CTCs in complete mesenchymal phenotype (44). This notion was further favored by a recent study showing that a complete EMT in breast cancer cells caused by loss of E-cadherin led to excessive oxidative stress and subsequently cell death, culminating in the decreased number of CTCs and reduced distant colonization (45).

However, it remains controversial which state (primarily epithelial with a moderate mesenchymal trait or primarily mesenchymal with moderate epithelial traits) plays a more crucial role in metastasis. Previously, it was revealed in human breast cancer specimens that elevated levels of mesenchymal CTCs during metastasis are correlated with acquired drug-resistance and poor prognosis (46). This was contrasted by recent studies showing that Lgr5, a mesenchymal marker (47), was negative in the majority of CRC CTCs (48). It was also reported that in breast cancer, CTCs with a dominating epithelial phenotype and modest mesenchymal features had the strongest metastatic potential among all identified phenotypes, and the proportion of epithelial CTCs was significantly related to distant metastasis and poor prognosis in patients (49). Overall, the determinants of metastasis occurrence may be the acquisition of features that make CTCs adaptive and viable, rather than certain fixed types of phenotypes.

#### Extravasation

During extravasation, CTCs lodge in narrow microvasculatures and protrude through the endothelial barrier at distant anatomical sites. EMT has been identified to facilitate CTCs extravasation through the opening of the endothelial barrier and alterations in CTCs shape and motility. In a drosophila CRC model, *in vivo* imaging visually demonstrated that overexpressing Snail in tumor cells triggered the breakdown and remodeling of the basal lamina to facilitate transendothelial migration (50). A previous study in zebrafish model also provided evidence suggesting that Twist upregulation in extravasating breast tumor cells induced reorganization of intercellular junctions of endothelial cells (51). What’s more, a recent study indicated that EMT activation of breast cancer cells orchestrated cytoskeleton rearrangement to endow cancer cells with polarized and aggressive morphology, thereby enhancing extravasation to form lung metastases (52).

The maintenance of EMT is considered more important as compared to the initiation of EMT, as the stroma-derived signaling that activates EMT in the microenvironment of primary lesion is absent throughout the vast majority of metastatic cascade. The epigenetic regulation of members of miR-200 family was acknowledged as key process of EMT maintenance in a variety of cancers (6, 22, 36, 53). A recent study in breast cancer using mathematical methods revealed the dynamic regulation of a negative feedback loop miR200s/Zebs, *via* which a short time exposure to stimuli was sufficient to sustain EMT for a long time (53). Moreover, data from single-cell resolution assay disclosed the epigenetic regulation of *miR-200* gene in CTCs. By focusing on the promoter methylation of *miR-200c/141*, *miR-200b/a/429*, and *CDH1* in CTCs isolated from patients with metastatic breast and prostate cancer, conspicuous heterogeneous methylation patterns were observed not only among patients, but also within the same individual. The observed heterogeneity supported the notion that CTCs reside in the dynamic transition of epithelial and mesenchymal axis (54). Besides, altered levels of miRNAs such as miR-16, miR-21, miR-31, and miR-210, which were identified relevant to EMT, were also observed in CTCs across a number of types of cancer (55).

In addition, it is believed that CTCs may interact with platelets through different cell surface receptors and ligands to sustain EMT *via* platelet-derived TGF-β signals (56) (**Figure 2**). Emerging evidence suggested that the downstream mechanism of this signal may involve epigenetic alterations. A recent study analyzed the methylation landscape in CTCs from patients with breast cancer and xenografts. Compared with single CTCs, CTC clusters showed higher hypomethylation for transcription factors binding sites, involving genes related to stemness, proliferation as well as cell adhesion which were also implicated in EMT (57–59) and this pattern was associated with poorer clinical outcomes (60). The authors concluded that the capacity of CTCs to spread in the circulation functions as the force to shape the DNA methylation pattern (60).

Recently, a hypothesis was put forward stating that EMT involved two tiers of control. While the acute phase was regulated by instant transcriptional regulation of EMT-TFs, a stable phase was enhanced by the global H3K36me2 levels, suggesting another possible epigenetic mechanism harnessed by cancer cells to maintain EMT (33). Overall, despite the technical challenges of investigating CTCs, the recent advance in single-cell sequencing has brought opportunities to study the epigenetic regulation of them, which may provide further insights into the dynamic molecular changes of EMT (61).

### EMT Reversal During Dormancy and Colonization

Upon reaching the distant sites, cancer cells need to adapt to the foreign microenvironment. While the vast majority of cancer cells will not survive, a small minority persist in a quiescence state as dormant cancer cells until reinitiate growth and form a colonization (**Figure 1**) Therefore, colonization is considered the major rate-limiting step in the metastasis cascade (2). EMT has been implicated in the regulation of dormancy by triggering cell cycle arrest. A substantial amount of evidences have shown that invasive cancer cells that have undergone EMT are in a state of low proliferation, that is, EMT-associated growth arrest (4). The underlying molecular mechanism involves the induction of cell cycle arrest by EMT TFs through directly suppressing cyclin expressions or inhibiting the expression of proliferating cell nuclear antigen (PCNA) (62, 63). While the mesenchymal state can exert proliferation inhibition on cells, the epithelial state allows growth. Clinical observation showed that most metastases across various types of carcinomas are highly differentiated (4). These clues lead to the proposition of the term MET, which assumes that invasive cells must re-differentiate to acquire the capacity of macrometastatic growth. Recent studies have demonstrated that MET is mainly triggered by contextual signals from microenvironment (**Figure 2**) (6, 64–66) as the absence of EMT-inducing signals from primary tumor microenvironment has already occurred after the early stage of metastatic cascade.

Indeed, the role of MET in metastatic outgrowth has been confirmed in numerous studies (10, 67–69). For instance, in squamous cell carcinoma, inhibition of Twist1 was required for MET to initiate tumor growth at a secondary site (68). In line with this notion, compelling evidence obtained *in vivo* has shown spontaneous interconversion between mesenchymal and epithelial phenotypes in primary tumors and metastases in breast and skin cancer, further reinforcing the existence and critical role of MET in reawakening proliferation (9, 10).

In addition to EMT maintenance, the negative feedback loops consist of pairs of miRNAs and EMT-TFs are also considered as the driving force underpinning the reversal of EMT. The most canonical feedback loops are miR-200-Zeb and miR-34-Snail (6, 22, 36, 53). Mathematical models analyzing the dynamics of EMT showed that these circuits resulted in epithelial states, mesenchymal states, or hybrid states. Once cells entered into an epithelial or mesenchymal state in response to environmental signals in the distant site, this state can be efficiently reinforced and self-stabled by feedback loops (70–72), ensuring phenotypic reversal. More importantly, compared with cells that directly switch to the extreme of the epithelial-mesenchymal axis, cells with an intermediated state can lower the threshold to initiate the epithelial or mesenchymal state, thus endowing partial EMT cells with higher plasticity (72).

Remarkably, both microRNAs and EMT markers in these tightly interconnected loops have also been shown to be elaborately regulated by epigenetic mechanisms encompassing DNA methylation and histone modification in different cancer types (22) (**Figure 3**). For example, in liver metastases of colorectal cancer, hypomethylation of *miR-200c and miR-141* promoter regions led to reversal of epithelial markers (73). In breast cancer, it was identified that H3K27me3-demethylase KDM6A-mediated bivalent chromatin state functioned as a regulator of a series of EMT/MET-associated bivalent genes. During EMT initiation, KDM6A was repressed, leading to transcriptional repression of epithelial genes, while during MET, the expression of KDM6A restored, enabling phenotypic reversal by reactivation of epithelial genes through H3K27me3 removal (74). Thus, collectively, the process of colonization on secondary sites involves regulation of MET that orchestrates a range of epigenetic regulations of cancer cells to enable a dynamic state shift, conferring adaptability to survive in distinct environments of target organs.

Noteworthy, the seeding patterns and dormant time differ substantially across different cancer types and subtypes due to the organ tropisms of metastatic cells (75). Under the influence of the target organs microenvironment, even tumors of the same type may exhibit diverse malignant behaviors due to differences among tumor subtypes. As an example, ER^+^ breast cancer often remains inactive in bone for decades, whereas triple-negative breast cancers tend to develop metastatic outbreaks in the lung within a short period of time (76). This heterogeneity is presumably due to distinct interactions between dormant malignant cells and the microenvironment, and the resulting context-dependent EMT-related epigenetic regulation. As the organ-specific patterns of metastatic colonization are being gradually unveiled recently, EMT-associated epigenetics in this process warrants further investigation.

## Therapies Targeting EMT *Via* Epigenetic Modification

### Targeting EMT in Different Stages of Metastasis

**Figure 4:**
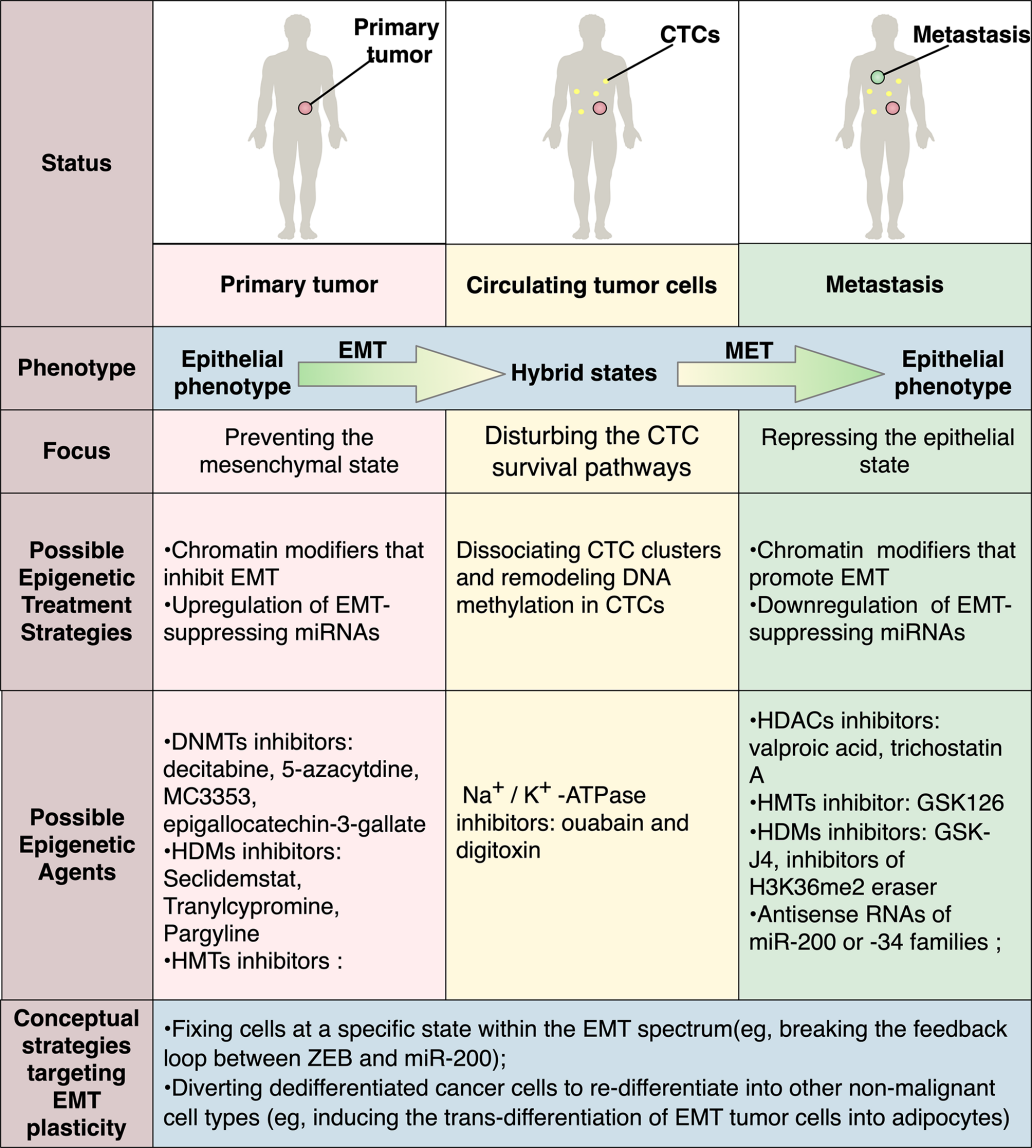
Possible therapeutic strategies targeting EMT plasticity via epigenetic modification. In different stages of metastasis, EMT and MET play contradictory roles, highlighting the need for context-specific therapeutic modalities for distinct disease status. As the interactions between EMT-TFs and epigenetic regulators have been widely revealed, targeting epigenetic mechanisms to manipulate EMT will have a promising therapeutic potential. Possible strategies include manipulation of miR-200s or miR-34 or chromatin modifiers that modulate EMT-TFs. Recently, treatments targeting CTCs are also emerging as exemplified by ouabain and digitoxin, which are FDA-approved Na^+^/K^+^-ATPase inhibitors and are identified to dissociate CTC clusters that harbor distinct DNA methylation pattern. Besides, various conceptual strategies targeting EMT plasticity are also proposed, with some of them are already proved effective in preclinical settings.

Owing to the reversible nature of epigenetic modification, it will be of prime interest to target epigenetic mechanisms to manipulate different cancer cell states (**Figure 4**). By far, major EMT-associated therapies aim at reverting or preventing EMT through targeting different aspects of this process, involving modulation of upstream pathways such as WNT, NOTCH, and TGF-β signaling (77), manipulation of stromal cells in the microenvironment such as tumor-associated macrophages, neutrophils and fibroblasts, employment of EMT-repressing miRNAs (78, 79), and direct inhibition of EMT-TFs *via* CRISPR–Cas9 (18, 80). As discussed above, the EMT-epigenetic network has been supported by a myriad of studies on the interactions between EMT-TFs and epigenetic regulators (22). Accordingly, approaches generally block EMT *via* epigenetic drugs targeting EMT-TFs, including DNMTs inhibitors, histone demethylase (HDMs) inhibitors, histone methyltransferases (HMTs) inhibitors and HDACs inhibitors.

Since piles of experiments have been done to prove the validity of epigenetic drugs for EMT in experimental models, these agents are being evaluated by or have gone through clinical trials, although the role of EMT is not specifically targeted and the involvement of EMT inhibition remains unknown (80–82). For instance, nucleoside analogs can inhibit the activity of DNMT1, resulting in hypomethylation, re-activation of the tumor suppressor gene, and EMT inhibition *in vitro*. As the DNMT1 inhibitors 5-aza-2’-deoxycytidine (decitabine) and 5-azacytdine have already been approved by FDA for treating acute myeloid leukemia and myelodysplastic syndromes, a clinical trial is underway to evaluate Aza-TdC for patients with advanced solid tumors (NCT03366116). A series of HDMs inhibitors and HMT inhibitors involving LSD1 inhibitor SP-2577 (Seclidemstat) (NCT03895684), EZH2 inhibitor CPI-1205 (NCT03525795), Tazemetostat (NCT01897571) are in trials against multiple cancer types. Moreover, many HDAC inhibitors such as Vorinostat, Tefinostat mocetinostat, and Panobinostat are also being extensively tested. Furthermore, as miRNAs have displayed critical roles in the regulation of EMT, the development of miRNA-based approaches has a promising potential. Progress has been made employing miRNAs in combination with multiple cytotoxic therapies, efficiently sensitize resistant cancer cells to chemotherapy in prostate cancer, pancreatic cancer and breast cancer (83–85).

Recently, treatments targeting CTCs in breast cancer were emerging as exemplified by ouabain and digitoxin, which have been approved by FDA as Na^+^/K^+^-ATPase inhibitors and were identified to dissociate CTC clusters that harbored a distinct DNA methylation pattern (60). Besides, it is noteworthy that while in most cases the inhibition of HDAC or HMTs resulted in repression of EMT and tumor growth, in some contradictory studies it showed precisely the opposite effect, again highlighting the context-dependent behaviors of epigenetic-regulated EMT networks and implying their possible role as MET blockers at the late stages of metastasis (86–88).

As discussed above, the H3K27me3-demethylase KDM6A, a bivalent chromatin regulator, served as the underlying mechanism of dynamic and reversible nature of EMT and MET in breast cancer cells, indicating that KDM6A may be a potential target to block the onset of MET. Indeed, treatment of KDM6A inhibitor GSK-J4 can repress various MET-associated genes in breast cancer cells (74). Furthermore, a recent study in pancreatic cancer revealed a unifying mechanism where a genome-wide change in H3K36me2 drove EMT and MET along the epithelial–mesenchymal spectrum. In this study, depletion of KDM2A, a histone demethylase that serves as the eraser of H3K36me2, locked tumor cells in a mesenchymal state and prevented MET, resulting in poor colonization of pancreatic cancer cells in the lung (33). Accordingly, it can be inferred that histone methyltransferases and demethylases that preferentially target H3K36me2 are potential targets for pharmacologically inhibition of MET at the end stage of tumor metastasis. Indeed, various epigenetic enzymes that target H3K36me2 already have small molecules inhibitors, among which some are currently being evaluated in clinical trials for different cancers, albeit further studies are required to clarify their specific roles in MET and metastatic colonization. However, given the distinct roles played by MET in the primary and metastatic lesions, it is worth noting that the true impact of epigenetic inhibitors in solid tumors could be confounding, for this reason, clinical trials of these therapies should not take alterations in primary tumor growth as the only outcome being evaluated.

### Targeting Epithelial–Mesenchymal Plasticity

As the states of cells undergoing EMT during metastasis cascade are found as a continuum across EMT spectrum, interventions can take advantage of this to disturb the transition which is required for metastatic seeding. Nevertheless, due to the currently limited ability to define whether there is a distant micrometastasis during the progression of cancer and the contradictory roles played by EMT across a series of scenarios, it is difficult to target the specific stage of cancer metastasis therapeutically, highlighting the risk of inappropriate intervention in EMT. More specifically, with the undetectable metastasis lesions, preventing the mesenchymal state may result in enhanced epithelial-associated colonization. On the contrary, repressing the epithelial state may promote the disassociation of cancer cells from primary tumor, underscoring the requirement for context-specific therapeutic modalities for primary site and metastatic lesion (18). Distinct from traditional systemic administration of chemotherapy drugs, recent advances in nanomedicine—which apply artificial nanomaterials with exceptional properties to pharmaceutical products—have held great promise to selective organ targeting drug delivery (89). As such, loading epigenetic drugs that target the specific states in the EMT spectrum within nanoparticles may contribute to the organ-exclusive therapeutic effect for primary versus metastasis tumors.

Despite this dilemma, the plastic nature of EMT still offers an opportunity for therapeutic targeting. Conceptually, the interventions targeting EMP involves either fixing cells at a specific state in the EMT spectrum to restrict their plasticity or breaking the interconversion process by trans-differentiation (**Figure 4**). Stabilization of the hybrid state, or in other words, restricting bidirectional transitions of EMP, is supposed to overcome the aforementioned side effects (4, 18, 80). Indeed, it was proposed by recent studies using subtle mathematical models that blocking the feedback loops integrated in the EMP network is likely to impair the metastatic potential of cancer cells (90). These feedback loops which enable the capability of cells to reversibly convert among a continuum of epithelial/mesenchymal states were mathematically proved to be a driving force behind phenotypic plasticity (90). Proof-of-principle validation of this idea was shown experimentally, where breaking the feedback loop between Zeb and *miR-200* by CRISPR/Cas9-based edition of Zeb1 binding sites in the promoter region of *miR-200s* reduced the metastatic potential of breast cancer *in vivo* (53). Alternatively, diverting dedifferentiated cancer cells to re-differentiate into other non-malignant cell types of the mesenchymal lineage could serve as a novel solution. Indeed, a seminal study exploiting EMP by inducing the trans-differentiation of EMT-state cancer cells into post-mitotic adipocytes has successfully reduced invasion and metastasis formation in a preclinical setting (91).

The above treatment options are built upon the notion that EMT/MET is reversible hence theoretically targetable. However, it should be noted that MET was not simply an antithesis of EMT, as different trajectories were used for cells that have undergone EMT(MET) to reverse back to the epithelial(mesenchymal) state (92, 93). Hence, the questions concerning the reversibility or irreversibility of EMT and/or MET have gained attention. Experimental studies have demonstrated that prolonged exposure to EMT stimulus such as TGF-β would cause irreversible EMT, locking cells in the stable mesenchymal state (53, 94, 95). So far, mechanisms regarding the concepts of when and if an EMT will be reversible have not been fully delineated.

Previously, a mathematical model integrating multiple feedback loops was proposed, in which the reversible transition from epithelial to hybrid phenotype was attributable to the miR-34-Snail feedback loop, followed by the conversion to the mostly irreversible mesenchymal state that was driven by the miR-200-Zeb1 feedback loop, moreover, the autocrine TGF-β-miR-200 feedback loop further stabilized the mesenchymal state to reinforce the irreversibility of EMT (96, 97). This model has been preliminarily validated in the human breast epithelial cell line MCF10A (98). Various attempts to characterize the underlying molecular mechanisms of the reversible or irreversible nature of EMT and/or MET have been made recently. These emerging studies have revealed global epigenetic alterations and identified major factors including Zeb1, RUNX2, GRHL, and CTCF involved in reversible or stabilized EMT/MET in multiple cell lines (69, 99–102). Besides, recent mathematical models proposed conceptually that epigenetic process may play a significant role in the irreversibility of EMT and MET, where epigenetic feedback-mediated suppression on miR-200 by Zeb1 led to the irreversibility of EMT, while epigenetic feedback-mediated suppression on Zeb1 by GRHL2 resulted in the irreversibility of MET (95, 103). Yet this irreversible MET has been proved to be unlocked by epigenetic modification in the preliminary experiment (95, 104). Future experimental work is still needed to uncover such mechanisms comprehensively.

## Perspective

EMT is a highly dynamic process involved in not only the metastatic cascade, but also several hallmarks of cancer, such as cancer cell stemness, therapy resistance, and immune evasion (5). These pleiotropic functions represent great therapeutic windows to reduce the risk of recurrence and enhance the efficacy of conventional chemotherapy or immunotherapy. However, many problems warrant further investigation (1). Although mounting evidence has indicated that instead of a binary process between epithelial and mesenchymal states, EMT represents different intermediate states, the specific context-dependent regulatory mechanisms underlying such partial states remain to be determined (2). To date, although emerging studies have demonstrated the existence of intermediate EMT *in vivo* using lineage-tracing strategies (9), most of which concerning the role of epigenetic mechanisms in EMT were conducted in cancer cells *in vitro*, and were merely restricted to the interaction between EMT-TFs and epigenetic regulators. To what extent do epigenetic modifications contribute to these hybrid states *in vivo*, especially during the distinct steps of the metastatic cascade, is still poorly understood. To address these two problems, future studies must take into consideration of the functional redundancy of EMT triggers and pathways and highly heterogeneous contexts across different cancer types (3). Moreover, the advance in techniques might help establish a clearer epigenetic-EMT network. At present, most studies employ epithelial markers such as EPCAM to isolate CTCs, which may yield confounding results due to the existence of partial EMT. Thus, the establishment of novel assays depending on the physical distinctions between CTCs and normal cells in the circulatory system will greatly facilitate the identification of precise and dynamic EMT of CTCs (105). From a therapeutic standpoint, many epigenetic reagents tested in current studies hold tremendous potential against cancer metastatic, further research is required to elucidate the underlying mechanisms by which epigenetic drugs regulate cell states transition *via* EMT modulation (4, 5) Given the complex and multifacet features of EMP, it has to be leveraged with spatiotemporal precision for therapy development, for instance, an EMT–MET map would help characterize the landscape of cells undergoing EMT/MET of tumor samples (92), thus facilitating selection of appropriate therapeutic targets.

## Author Contributions

H-NC and Z-GZ designed the article. Q-LL performed the literature search. Q-LL and ML drafted the work. H-NC and CH revised the work. All authors contributed to the article and approved the submitted version.

## Funding

This work was supported by (1) National Natural Science Foundation of China (81702378, 82073246, and 81821002); (2) China Postdoctoral Science Foundation (2019T120845, 2018M643496); (3) Sichuan Science and Technology Program (2019YFS0263); (4) Post-Doctor Research Project, West China Hospital, Sichuan University (19XJ0075, 2018HXBH007); and (5) 1.3.5 project for disciplines of excellence, West China Hospital, Sichuan University (2016105).

## Conflict of Interest

The authors declare that the research was conducted in the absence of any commercial or financial relationships that could be construed as a potential conflict of interest.
